# TGF*β*1, SNAIL2, and PAPP-A Expression in Placenta of Gestational Diabetes Mellitus Patients

**DOI:** 10.1155/2024/1386469

**Published:** 2024-07-30

**Authors:** Yujing He, Xiyao Yang, Na Wu

**Affiliations:** ^1^ Department of Endocrinology Shengjing Hospital of China Medical University, Shenyang 110004, China; ^2^ School of Life Science Liaoning University, 66 Chongshan Middle Road, Shenyang 110036, China; ^3^ Department of Pediatrics Shengjing Hospital of China Medical University, Shenyang 110004, China

**Keywords:** bioinformatics analysis, gestational diabetes mellitus, PAPP-A, SNAIL2, TGF*β*1

## Abstract

**Background:** Gestational diabetes mellitus (GDM) is a pregnancy-related diabetic condition that may cause serious complications. However, its pathogenesis remains unclear. Placental damage due to GDM may lead to several health issues that cannot be ignored. Thus, we aimed to identify the mechanisms underlying GDM by screening differentially expressed genes (DEGs) related to vascular endothelial cells in the GDM databases and verify the expression of these DEGs in the placentas of women afflicted by GDM.

**Methods:** We used GDM microarray datasets integrated from the Gene Expression Omnibus (GEO) database. Functional annotation and protein–protein interaction (PPI) analyses were used to screen DEGs. Placental tissues from 20 pregnant women with GDM and 20 healthy pregnant women were collected, and differential gene expression in the placental tissues was verified via qRT-PCR, western blotting, and immunofluorescence.

**Results:** Bioinformatics analysis revealed three significant DEGs: *SNAIL2*, *PAPP-A*, and *TGFβ1*. These genes were all predicted to be underexpressed in patients with GDM. The results of qRT-PCR, western blot, and immunofluorescence analyses indicated that SNAIL2 and PAPP-A in the placenta tissue of patients with GDM were significantly underexpressed. However, TGF*β*1 in the placenta tissues of GDM was significantly overexpressed.

**Conclusion:** SNAIL2, TGF*β*1, and PAPP-A may affect the placentas of pregnant women with GDM, warranting further investigation.

**Summary**

• SNAIL2 and PAPP-A were significantly underexpressed while TGF*β*1 was significantly overexpressed in the placenta of pregnant women diagnosed with GDM, warranting further investigation.

## 1. Introduction

Gestational diabetes mellitus (GDM) is defined as carbohydrate intolerance observed for the first time during pregnancy [1–3]. The prevalence of GDM, a common disorder that manifests during pregnancy, is increasing worldwide and has been attracting the attention of the scientific community [4]. Its incidence rate in China is as high as 8.4%–18.9% [5]. Risk factors for GDM include maternal weight and obesity, advanced age, history of GDM, and family history of Type 2 diabetes [6, 7]. Poorly regulated blood glucose levels of pregnant women not only increase the risk of maternal pre-eclampsia (PE) and premature delivery but also lead to spontaneous abortion, malformation, hypoxia, and in severe cases, intrauterine death. Hyperglycemia often leads to fetal macrosomia and increases the probability of dystocia. Newborns are prone to complications, such as neonatal respiratory distress syndrome, hypoglycemia after birth, and even death in severe cases [3]. GDM exerts an adverse effect on long-term blood glucose and lipid levels of mothers and offspring, endangering life and health in severe cases [8, 9]. GDM is a complex disease; hence, its pathogenesis remains to be fully clarified and warrants further investigation.

The placenta plays an essential role in the regulation of metabolic function and material exchange between a pregnant woman and the fetus [10–14]. Continuously high glucose levels and oxidative stress associated with GDM may damage the placenta. This damage may cause placental dysfunction, which leads to many functional changes, such as impaired regulation of vasodilation and vasoconstriction, impeded or excessive angiogenesis, decreased barrier function, and increased inflammation [14, 15]. Some studies have suggested that the functioning of placental vascular endothelial cells is impaired in patients with GDM [16, 17] and that changes in the insulin signaling pathway may lead to endoplasmic reticulum stress [18, 19], aberrant angiogenesis [20, 21], and lipid metabolism [22–24]. PE is a common complication of GDM, with risk factors similar to those of GDM, including obesity and advanced age [25]. Moreover, it has been established that both risk factors may cause vascular endothelial cell dysfunction [26, 27].

With the rapid advancement of high-throughput microarray hybridization and sequencing technology, the amount of publicly available data pertaining to various nucleic acid sequences has increased rapidly. The Gene Expression Omnibus (GEO) is currently the largest open gene expression database that can be used to obtain gene expression profile data for most species. Therefore, screening genes related to GDM and predicting and verifying their functions via relevant databases may help elucidate the mechanism(s) underlying GDM. We screened the differentially expressed genes (DEGs) related to vascular endothelial cells in the GDM database. In addition, we verified the expression of these DEGs in the placenta. In the current study, our purpose was to explore the cellular processes underlying GDM in an attempt to identify new mechanisms.

## 2. Methods

### 2.1. Chip Data Screening and Acquisition

We collated GDM-related chip data that had been uploaded to the GEO database of the National Center for Biotechnology Information (NCBI) before October 2021, through PubMed, using “gestational diabetes mellitus” and “placenta” as keywords, and setting “Homo sapiens” as the species. In summary, the chip data were screened according to the requirements of this study, and the gene chips related to GDM were screened according to expression profiling by the array.

### 2.2. Screening of DEGs

Collected datasets were screened and analyzed using the online analysis software, GEO2R, provided by the GEO database. DEGs in each database were screened using *p* < 0.05 and |logFC| > 1.5 as criteria; upregulation and downregulation of gene expression were determined according to the positive and negative value of logFC, respectively. Further, the differential genes highly related to the function of vascular endothelial cells were screened by consulting the literature.

### 2.3. Functional Annotation and Enrichment Analysis

DAVID (http://david.ncifcrf.gov/) was used to conduct Gene Ontology (GO)—and Kyoto Encyclopedia of Genes and Genomes (KEGGs)—pathway-related analyses of screened DEGs. GO includes three categories: biological processes, cellular components, and molecular functions. It categorizes DEGs according to their molecular functions and biological processes. KEGG mainly focuses on the functional enrichment of signaling pathways involving DEGs. A protein–protein interaction (PPI) network was constructed and analyzed using DEGs inputted into STRING (http://string.embl.de/). COREMINE (http://www.coremine.com/medical) was used to conduct a literature co-occurrence analysis using “gestational diabetes mellitus,” “preeclampsia,” and DEGs.

### 2.4. Patients' Samples and Tissue Preparation

Singleton pregnant women who had undergone cesarean sections and been closely followed up postpartum at Shengjing Hospital, affiliated with China Medical University, were enrolled in this study from December 2020 to August 2021 (Table 1). GDM was diagnosed using an oral glucose tolerance test (OGTT). All subjects were orally administrated 75 g of glucose, and blood glucose levels were measured at 0, 1, and 2 h. Those with fasting blood glucose > 5.1 mmol/L, 1 h postprandial blood glucose > 10.0 mmol/L, or 2 h postprandial blood glucose > 8.5 mmol/L were categorized as suffering from GDM. Based on the results of the OGTT, which involved administering 75 g of glucose during pregnancy, the participating women were divided into two groups as follows: a normal glucose tolerance group (*n* = 20) and a GDM group (*n* = 20). None of the study participants had a history of pregestational Type 1 or Type 2 diabetes, hyperthyroidism, Cushing's syndrome, pancreatitis, other diseases affecting blood glucose levels, or pregnancy-related complications, such as PE or severe heart and liver damage. All experimental procedures were approved by the Medical Ethics Committee of Shengjing Hospital, affiliated with China Medical University (2021PS338K). Informed consent was obtained from all participants. Placental tissue with a size of 1 cm^3^ was collected from the maternal surface of the placenta 2–3 cm away from the umbilical cord and frozen in liquid nitrogen to extract total RNA and protein.

### 2.5. Detection of Differential Gene Expression in Placenta by qRT-PCR

Total RNAs from the placenta were isolated using the TRIzol reagent (Thermo, Massachusetts, USA). The expression levels of DEGs were detected via qRT-PCR using a HiFiScript cDNA Synthesis Kit and an SYBR Mixture (CWBIO, Beijing, China), according to the manufacturer's instructions. Primers were synthesized by GENEWIZ WEEK (Suzhou, China). Each sample was analyzed in triplicate. All data calculations are based on GAPDH as internal reference to calculate relative expression. Data from more than three biological replicates were subjected to final quantitation and statistical analysis using the 2^-△△Ct^ method. The primer sequences used for qRT-PCR analysis are listed in Table 1.

### 2.6. Detection of Differential Gene Expression in Placenta by Western Blot

Extracted proteins were boiled in sodium dodecyl sulfate-polyacrylamide gel electrophoresis protein loading buffer (Beyotime, Shanghai, China). Thirty micrograms of protein was run on a 5%–8% gradient polyacrylamide gel and transferred to PVDF membranes (Millipore, Billerica, MA, USA).

The membranes were sliced and incubated with mouse anti-SNAIL2 monoclonal antibody (Abcepta, San Diego, USA, 1 : 500), rabbit anti-TGF*β*1 polyclonal antibody (Abcepta, San Diego, USA, 1 : 500), rabbit anti-PAPP-A polyclonal antibody (Abcam, Cambridge, MA, USA, 1 : 1000), or rabbit anti-*β*-tubulin polyclonal antibody (Proteintech, Rosemont, USA), overnight at 4°C. Next, the membranes were incubated with horseradish peroxidase (HRP)-conjugated goat anti-rabbit IgG or goat anti-mouse IgG (Elabscience, Wuhan, China, 1 : 5000) for 2 h at 37°C. The membranes were then incubated with ECL reagents for 5 min (Millipore, Billerica, MA, USA) and exposed to X-ray film (Kodak, USA); *β*-tubulin was used as the loading control. The experiment was repeated at least thrice. Optical densities of the bands were analyzed using a gel image-processing system (Gel-Pro Analyzer software). Protein level was represented as the relative ratio of DEG signals to *β*-tubulin signals.

### 2.7. Detection of Differential Gene Expression in the Placenta by Immunofluorescence

Each specimen was placed on a wax block and sliced into 5 *μ*M slices using a slicer, which were then unfolded in a warm water dish. Next, the expanded tissue slices were moved onto a glass slide, placed in a 60°C incubator for 4 h, and dried. The slices were then dewaxed in water, subjected to antigen repair, and blocked using goat serum. Next, the slices were incubated overnight (at 4°C) with CD31 rabbit antibody (Abcepta, San Diego, USA, 1 : 50) and other DEG antibodies as described previously. After washing thrice, the cells were incubated with Alexa Fluor 488 labeled goat anti-rabbit IgG (H + L) (Beyotime, China, 1 : 400) and Cy3 labeled goat anti-mouse IgG (H + L) (Beyotime, China, 1 : 400) for 1 h. The sections were washed again with PBS and incubated with DAPI (Solarbo, China, 1 : 500) for 10 min. Finally, the sections were washed thrice with PBS, observed, and photographed under a microscope.

### 2.8. Statistical Analyses

Data are presented as means ± standard deviations (SDs); SDs are represented by error bars. Comparisons between the two groups (paired and unpaired) were performed using a two-tailed Student's *t*-test. One-way analysis of variance (ANOVA) and Tukey's post hoc test were used for comparisons involving more than two groups. Statistical significance was set at *p* < 0.05. All statistical analyses were performed using SPSS software (version 16.0).

## 3. Results

### 3.1. Data Collection

A total of 879 eligible datasets in the GEO database were screened. After screening, one set of microarray data that met our standards was selected as follows: GSE87295 (https://www.ncbi.nlm.nih.gov/geo/query/acc.cgi?acc=GSE87295).

GSE87295, which contains human umbilical vein endothelial cell transcriptome sequencing data using the GPL10558 platform, was submitted by Ajith et al. The GDM group consisted of five pregnant women with GDM (*n* = 5) while the normal group consisted of five pregnant women without GDM.

### 3.2. Screening of DEGs

**Figure 1:**
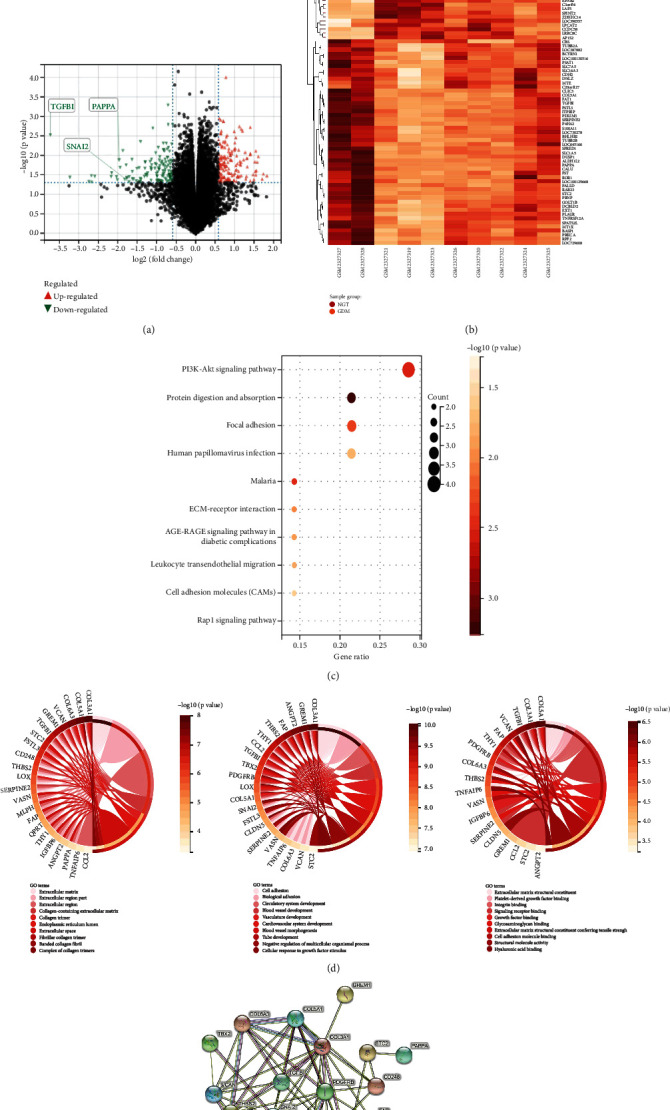
Function and pathway analyses for DEGs. (a) TGF*β*1, PAPP-A, and SNAIL2 were predicted to be underexpressed in data analysis. (b) Heat map of DEGs. (c) Significant pathways associated with DEGs. (d) Gene Ontology annotation of DEGs. (e) PPI network of DEGs.

**Figure 2:**
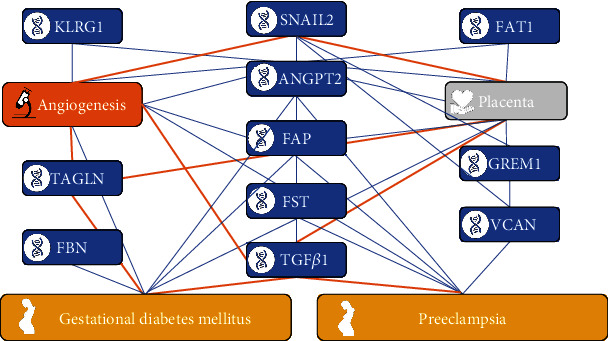
Literature co-occurrence analysis.

The GEO2R software tool provided by the GEO database was used to screen DEGs in the GSE87295 dataset. Thus, 49 DEGs were screened in the HUVEC group, using *p* < 0.05 and |logFC| > 1.5 as criteria. The DEGs in the database were mainly located in the extracellular and matrix regions. Their main molecular functions included protein and glial binding, which in turn are associated with the biological processes of cell adhesion and angiogenesis. Moreover, the KEGG pathways were not enriched, similar to the DEGs in the other two groups, which may be related to the small number of DEGs. Different genes in each group were enriched, as shown in Figures 1 and 2.

### 3.3. PPI Network of DEGs, Literature Co-Occurrence, and Key Gene Screening

Preliminary construction of the PPI network and the literature co-occurrence network of DEGs enabled us to select DEGs in the GSE87295 dataset and determine the relationship between GDM and important DEGs. We used “gestational diabetes mellitus,” “preeclampsia,” and key DEGs for literature co-occurrence analysis. Angiogenesis, which is mostly observed in the placenta, is an important biological process associated with these genes. Literature-based comparisons and initial data analyses were used to select key genes. Having given due consideration to all aspects, we selected *SNAIL2*, *TGFβ1*, and *PAPP-A* as the key genes.

### 3.4. Detection of Differential Gene Expression in Placenta

There were no significant differences between the general data of pregnant women in the GDM and control groups (Table 2).

Subsequently, qRT-PCR indicated that the expression levels of TGF*β*1 in the GDM group were higher than those in the control group, while the expression levels of PAPP-A and SNAIL2 in the GDM group were lower than those in the control group (*p* < 0.05).

Western blot results demonstrated that expression levels of TGF*β*1 in the GDM group were higher than those in the control group, while the expression levels of PAPP-A and SNAIL2 in the GDM group were lower than those in the control group (*p* < 0.05).

Our immunofluorescence results also confirmed that the expression levels of TGF*β*1 in the GDM group were higher than those in the control group, while the expression levels of PAPP-A and SNAIL2 in the GDM group were lower than those in the control group (*p* < 0.05).

**Figure 3:**
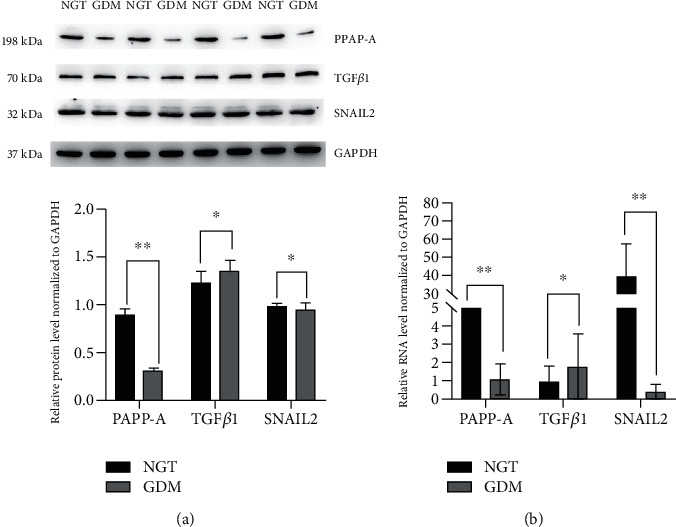
Detection of DEG expression in the placenta (*n* = 20). (a) Western blot analysis of the DEG levels in the placentas of GDM patients or normal patients. The DEG levels were quantified as shown. (b) qPCR analysis of DEG levels in the placentas of GDM or normal patients; bars = 20 mm. All data are presented as means ± SEM of 20 replicates; ∗*p* < 0.05, ∗∗*p* < 0.01 compared with the indicated group.

**Figure 4:**
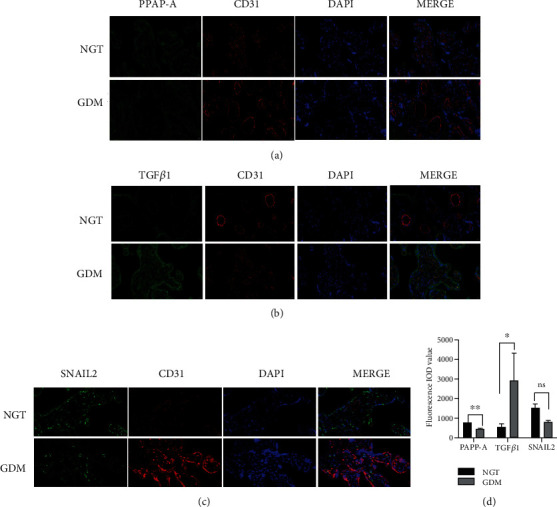
Detection of DEG expression in the placenta via immunofluorescence. (a) Immunofluorescence double staining of PAPP-A and CD31 in the paraffin sections of two groups. (b) Immunofluorescence double staining of TGF*β*1 and CD31 in paraffin sections of two groups. (c) Immunofluorescence double staining of SNAIL2 and CD31 in the paraffin sections of two groups. (d) Fluorescence intensity analysis of DEG levels in the placentas from two groups; bars = 20 mm. All data are presented as means + SEM of 20 replicates; ∗*p* < 0.05, ∗∗*p* < 0.01 compared with the indicated group.

The results are shown in Figures 3 and 4.

## 4. Discussion

GDM is a condition commonly observed in pregnancies during clinical practice. The incidence of GDM differs according to region and age group; however, its exact pathogenesis remains unclear. Evidently, its pathogenesis is associated with an increase in the number of gestational weeks, which causes anti-insulin-like substances, such as placental lactogen, estrogen, progesterone, cortisol, and placental insulin enzymes, to accumulate during midpregnancy and late pregnancy, causing insulin sensitivity of affected women to keep declining with increasing gestational weeks, resulting in an increase in their blood glucose levels [19]. We screened the differential genes (TGF*β*1, SNAIL2, and PAPP-A) by bioinformatics analysis of datasets combined with literature retrieval of differential genes and keywords such as GDM, vascular function, and endothelial cells.

SNAIL2, which encodes a transcription factor with a zinc finger structure, acts as a key regulator of epithelial–mesenchymal transformation (EMT). SMAD-interacting protein-1 competitively binds to E-box in the promoter region of the gene encoding E-cadherin. SNAIL2 transforms epithelial cells into mesenchymal cells by directly inhibiting the expression of E-cadherin, which promotes EMT [28]. SNAIL2 has previously been described as a factor involved in cancer. It has been found that different mechanisms of E-calcium mucin inactivation occur in most breast cancer tumors. A significantly negative correlation was observed between high SNAIL expression and decreased or downregulated E-cadherin expression. Our results indicated that SNAIL2 expression in the GDM placenta is decreased, indicating that EMT in the placenta is inhibited during GDM, which in turn impacts the proliferation of trophoblasts into vascular endothelial cells and affects the function of vascular endothelial cells.

The TGF-*β* family is involved in many biological processes, such as cell proliferation, differentiation, and death [29]. TGF*β*1 enables the pancreas to maintain homeostasis and also plays a physiological role similar to that of insulin. It is a key cytokine involved in insulin resistance and obesity [9] and is differentially expressed in the human endometrium and placenta [29, 30]. Our results showed that TGF*β*1 expression was upregulated in the placenta of GDM patients. TGF*β*1, an important factor that promotes endothelial cell differentiation during angiogenesis, may be one of the mechanisms underlying “hypervascularization” of the placenta of GDM patients.

PAPP-A is mainly located near actin filaments in the cytoplasm. PAPP-A, an actin-binding protein, promotes actin crosslinking [31–33] via globular actin polymerization and participates in the regulation of cytoskeletal rearrangement [34]. Its function is associated with cell migration and angiogenesis [35, 36]. Studies have indicated that PAPP-A may promote the elongation of vascular endothelial cells. The PI3K Akt and mTORC1 pathways are also associated with vascular endothelial growth factor A [37]. However, our study indicated that the change in PAPP-A expression was not significant, which finding contradicts the results of Varberg et al. [34]. The reason for this discrepancy warrants further exploration.

Bioinformatic data mining allows relevant gene chips and sequencing data to be screened and analyzed along different research directions, thereby providing novel insights into the diagnosis and treatment of clinically important diseases, as well as preliminary research aimed at these diseases, and related research of Du et al. and Sun et al. in the field of placental dysfunction in GDM stands out [38, 39].

In this study, we screened transcriptome datasets of patients with GDM and analyzed DEGs. These DEGs and proteins were mainly located in the extracellular spaces and regions. Their main molecular functions were described as polymerization, platelet-derived growth factor, hyaluronic acid, and transforming growth factor *β*. These proteins are primarily involved in cell adhesion and angiogenesis. KEGG pathway analysis showed that the DEGs were mainly involved in ECM receptor interactions, adhesion, and the PI3K Akt pathway, among others. Among them, *SNAIL2*, *TGFβ1*, and *PAPP-A* were predicted to be underexpressed in GDM and play an important role as key genes in the GDM network. Concurrently, western blotting and qRT-PCR also demonstrated that SNAIL2, TGF*β*1, and PAPP-A were differentially expressed between GDM patients and healthy pregnant women. This may be related to the younger phenotypes of vascular endothelial cells and is worthy of future attention. Our verification of these DEGs indicated that the expression and prediction of SNAIL2 were identical.

Therefore, *SNAIL2*, *TGFβ1*, and *PAPP-A*, which are significantly differentially expressed in the placenta of patients with GDM, may play an important role in the occurrence and development of GDM, via the exertion of adverse effects on the proliferation of vascular endothelial cells and the adhesion and proliferation of smooth muscle cells. Although some preliminary findings have been made in this study, and the expression of differential genes has been preliminarily verified by tissue verification, we also have to admit that there are some limitations in the study. The lack of further functional verification and mechanism research may limit the accurate explanation of the biological significance of differential genes. Therefore, the future research still needs to be further expanded, including carrying out functional experiments and exploring potential mechanisms. We hope that the discussion of these limitations will attract more researchers' attention and promote the in-depth exploration and understanding of this field. Through continuous efforts, we believe that we can more comprehensively reveal the mechanism of differential genes in the process of vascular dysfunction in GDM and provide more in-depth theoretical support for the diagnosis and treatment of vascular complications in GDM.

## Figures and Tables

**Table 1:** Primer sequence.

**Gene**	**Sequence (**5′–3′**)**	**Primer length (bp)**
GAPDH.F	AGGTCGGTGTGAACGGATTTG	21
GAPDH.R	GGGGTCGTTGATGGCAACA	19
TGF*β*1.F	GGCCAGATCCTGTCCAAGC	22
TGF*β*1.R	GTGGGTTTCCACCATTAGCAC	21
PAPP-A.F	ACAAAGACCCACGCTACTTTTT	22
PAPP-A.R	CATGAACTGCCCATCATAGGTG	22
SNAIL2.F	CGAACTGGACACACATACAGTG	22
SNAIL2.R	CTGAGGATCTCTGGTTGTGGT	21

**Table 2:** Basic characteristics of subjects.

**Clinical parameters**	**Control (** **N** = 20**)**	**GDM (** **N** = 20**)**
Mean of age (Y)	31.238 ± 2.300	30.826 ± 2.741
Mean of height (cm)	164.00 ± 4.889	161.913 ± 4.430
Mean of weight (kg)	75.286 ± 7.927	75.752 ± 6.410
Prepregnancy BMI (kg/m^2^)	22.275 ± 2.582	22.930 ± 3.813
Pregnancy weeks	36.762 ± 3.897	36.304 ± 2.653
The number of abortions	0.905 ± 0.831	0.609 ± 0.722
Basic systolic BP (mmHg)	76.905 ± 6.610	78.348 ± 6.997
Basic diastolic BP (mmHg)	118.810 ± 8.600	118.826 ± 6.719

## Data Availability

The datasets used in this study can be found in Gene Expression Omnibus (GEO) (GSE87295 [https://www.ncbi.nlm.nih.gov/geo/query/acc.cgi?acc=GSE87295]). Please refer to the Data Availability section of the Author's Guidelines for more details.
